# Supplementary material for: Patterns of Intron Gain and Loss in Fungi
Source: PLoS Biol. 2004 Nov 30;2(12):e422. doi: 10.1371/journal.pbio.0020422 (PMC532390; doi:10.1371/journal.pbio.0020422)
Supplement: Table S1 — Also available at http://genes.mit.edu/NielsenEtAl/. (4.3 MB ZIP). [file pbio.0020422.st001.zip › NielsenEtAl/html/379.html]

AN3422.1.NCU04612.1.MG00800.1.FG09903.1


```
 CLUSTAL W (1.82) Multiple Sequence Alignments - Introns Inserted


Sequence 1: MG00800.1	523 aa
Sequence 2: FG09903.1	518 aa
Sequence 3: NCU04612.1	511 aa
Sequence 4: AN3422.1	539 aa
Alignment Length: 557 aa
Number Identitical Residues: 236 aa
Alignment Score (without introns) 11596


MG00800.1 	MADPFAPRTMKRRNVKGLALTPAAPKPPPTAENAPIHR-------DSDQHAQLEIGIEFN
NCU04612.1	MADPFAPRSMKRKNVKGLALAPAAPKPPPTAETFYKDK-------DDDNNAQLEIGIEFN
FG09903.1 	MSDPFAPRSMKRKNVKGLALKAAAPRPPPTAETSNHEYS---GSQDAGRDEQLEIGIEYK
AN3422.1  	MADQFKARTLKRKNVKGLALN-AAPKSTSTLSNGDSQVPGAAGNGESNRTDTLEIGLEFR
          	*:* * .*::**:*******  ***:...* ..   . ..::.. : ..   ****:*:.

MG00800.1 	LDLRPEDLEVIKDLGSGNGGTVSKVRHIPTNTVMARK0VIHVEAKREMRKRIVRELQIMH
NCU04612.1	LDLRPEDLDLIKELGAGNGGTVSKVRHIPTNTVMARK0VIHVEAKKEMRKRIVRELQIMH
FG09903.1 	LDLRPEDLEILKELGSGNGGTVSKVKHITTGTVMARK0VIHVEAKREIRKRIVRELQIMH
AN3422.1  	LDLRSEDLITLKELGAGNGGTVSKVMHASTKVVMARK0IIRVDAKENVRKQILRELQVGH
          	****.***  :*:**:********* * .* .***** :*:*:**.::**:*:****: *

MG00800.1 	SCNSEYIVTFYGAFLNENNDVIMCMEYADVG2SLDRVSRVFGPIRVDVLGKIAEATLGGL
NCU04612.1	GCHSEYIVTFYGAFLNEHNDVIMCMEYMDVG2SLDRISRVFGPVRVDVLGKIAEATLGGL
FG09903.1 	GCHSDYIVTFYGAFLTPNNDVIMCMEYMDVG2ALDRVSRVFGPVRVDVLGKIAEATLGGL
AN3422.1  	DCNSPNIVTFYGAFQNEARDIVLCMEYMDCG2SLDRISKDFGPIRVDVLGKITESILAGL
          	.*:*  ******** .  .*:::**** * * :***:*: ***:********:*: *.**

MG00800.1 	TYLYAKHHIMHRDIKPSNILVNSRGSIKLCDFGVSGELINSIADTFVGTSTYMAPERIQG
NCU04612.1	TYLYSKHHIMHRDIKPSNILVNSKGHIKLCDFGVSGELVNSVADTFVGTSTYMAPERIQG
FG09903.1 	TYLYTKHHIMHRDIKPSNILVNSRGGIKLCDFGVSGELVNSIADTFVGTSTYMAPERIQG
AN3422.1  	VYLYEAHRIMHRDIKPSNVLVNSRGNIKLCDFGVATETVNSIADTFVGTSTYMAPERIQG
          	.***  *:**********:****:* ********: * :**:******************

MG00800.1 	EKYTVKSDVWSFGLSIMELAIGKFPFAASEQLS--DAESAPAGILDLLQQIVHEPAPKLP
NCU04612.1	DKYTVKSDVWSFGLTVMELAIGKFPFASDQLGD--DAENAPAGILDLLQQIVHEPAPKLP
FG09903.1 	EKYTVKSDVWSFGLSIMELAIGKFPFAASEQVS--DGDFAPAGILDLLQQIVHEPAPKLP
AN3422.1  	GAYTVRSDVWSVGLTVMELAVGRFPFDTTDSAAGDRASAGPMGILDLLQQIVHEPAPKLP
          	  ***:*****.**::****:*:*** : :   .. .. .* ******************

MG00800.1 	KSDAFPQILEDMIQKCLYKNPDDRPTPEELF0ERDPFVQAAKRTPVDLREWAFGLMERDN
NCU04612.1	KSDAFPQILEDMIQKCLYKEPERRPTPQELF0DRDPFVQAAKRTPVDLREWACSLMDRDN
FG09903.1 	KSDAFPSILDDMIQKCLYKEPERRPTPQELF0DRDHFVQAAKRTPVDLREWAVGMMERDN
AN3422.1  	RSDAFPPVLHEFVAKCLLKKSEERPTPLELY0EKDAFLAAAKRTPVDLQEWAISMMERHN
          	:***** :*.::: *** *:.: **** **: ::* *: *********:*** .:*:*.*

MG00800.1 	RKSHLAPQLSPATADLLRSSDSPT----ATYHGDDRP---LETPTSGEIPILGA------
NCU04612.1	RKSHLQPQLSPSSHDFLRSSDEPT-------------------PTSGEIPILGT------
FG09903.1 	RKSHLAPQLSPSTQDLLRSSDSPTQSQQAQAQAQAQPEERSHTPTSGEIPIGGAG-----
AN3422.1  	RKSYLAPPAPKSLKETRESPSPAQAPSPVQKHGTSRP----SRGTTGEIPLNVARDSSSH
          	***:* *  . :  :  .*.. .  ..     .   .       *:****:  : .::: 

MG00800.1 	--IVSPRDQ--YGSISSRSPTRAGGGIGGLGRPGGSALHPGLGPRMATTSSVP----MSS
NCU04612.1	--MVSPRDQ--HGSVPTRPPPSSRTGSA--GRTGGPSVHPGLGSRAATTGSQPRYPQQAP
FG09903.1 	--ITSPRDQ--YGSNQNRSPPRNGHASS--RTP--ASAHPGLGSRVITTNSIP----KVS
AN3422.1  	QRQHAPQSQSHFSSNPSHYSSNSSHYSSRSSRSSPPISLEHLSLESKQDEHRPTR--RPS
          	.   :*:.*: ..*  .: ..      .    .. .     *. .       *      .

MG00800.1 	GASYHGVSSSSADYASAIPAQPSSASKATFS-LPMRPAPAG-TIPPPPPRKE-TPDESKK
NCU04612.1	PPPHPPFEKRPSMSTGHSYTSSSSSSRSGFA-LPIRPGPPGQPMPPPPPRRQLTAEELQR
FG09903.1 	GYPDN--------------VGPVSANAATFS-LPVRPAPPGGPLPPPPPRKE-TPDELRR
AN3422.1  	RTPLGDSSSSLDQSLRPSIGSRSASSHNTNSRMPLQSTALPFRVAPTPGGYP-TPNSTSW
          	  .    ..              ::.    : :*::. .    :.*.*     *.:.   

MG00800.1 	ESRRQALFGPPSSTGSYGF-
NCU04612.1	ESRRQAAMQPPTGRF-----
FG09903.1 	ENRRQATFGLPPNP-SYGIQ
AN3422.1  	Q-RRGDHMGAL---------
          	: **   :
```
